# An innovative protocol to increase egg production of chicken layers

**DOI:** 10.1371/journal.pone.0305099

**Published:** 2024-06-06

**Authors:** Furkan Alaraji

**Affiliations:** Department of Pathology and Poultry Diseases, University of Kufa, Kufa, Al-Najaf Province, Iraq; Ain Shams University Faculty of Agriculture, EGYPT

## Abstract

This study investigated the effects of different doses of limestone, light durations, light intensities, and vitamins on both the productive performance and egg quality. The study utilized two rearing houses (control and treatment), each accommodating 75000 Lohmann Brown Classic chicks reared in open-sided rearing cages from one day old until they reached 89 weeks of age. Throughout the laying period, the hens were subjected to a specific light regimen (light = 14 h; dark = 10 h a day). At the end of experiment, the treatment group displayed significant (*p*<0.05) differences compared to the control group across various parameters. Notably, the treatment group exhibited lower daily feed intake (treatment: 112 g/bird vs control: 115 g/bird), 9.6% higher egg production (treatment: 78.5% vs control: 68.9%), lower body weight (treatment: 2057 g vs control: 2073 g), lower feed conversion ratio (FCR)/egg (treatment: 1.44 vs control: 1.69), higher egg weight (treatment: 69.4 g vs control: 68.5 g), greater egg mass (treatment: 56.14 vs control: 48.76), greater shell thickness (treatment: 3.52 mm vs control: 3.44 mm), and greater shell weight (treatment: 9.3 g vs control: 8.79 g). However, the albumin weight, yolk weight, yolk diameter, shape index, and Haugh units (HU) were not significantly (*p*˃0.05) affected after 75 weeks of treatment when compared with those of the control group. Therefore, this study is the first of its kind to demonstrate that different ratios of limestone, different durations and intensities of light, and different vitamin supplementation doses in the treatment group (subjected to the novel rearing recommendations described in this study) may yield a profit of 180,541 USD, exceeding the baseline profit of the control group (subjected to conventional rearing methods).

## Introduction

In recent decades, the egg layer-raising industry has undergone substantial transformation and shown extraordinary resilience in the face of unprecedented upheavals. The poultry sector’s advancement can largely be attributed to increased egg production, enhanced feed efficiency, and improved egg quality to meet market demands. As the annual egg demand rises by 1 million tons, with the current egg demand reaching 75 million tons, there is an urgent need for management strategies to maximize genetic potential. For example, to achieve a target egg mass of 20 kg per layer from 20 to 76 weeks of age, a minimum of 50 million layers will need to be recruited annually to the meet rising demands [1].

Although the global human population is predicted to rise to 9.7 billion by 2050, marking an increase of approximately one-third from the 2015 level, many countries still continue to have alarmingly high rates of different forms of malnutrition [2].

Europe consumes over 100 billion eggs annually, as eggs are prized for their excellent nutritional value and versatility as an ingredient across various cuisines. Furthermore, advancements in animal welfare standards and European legislation have resulted in the remarkable evolution of egg production techniques over the last 15 years [3].

Multiple studies have reported that hen egg output and quality are affected by both genetic and environmental variables [4]. Heritability calculations suggest that genetic variables account for 30–70% of phenotypic variation, while environmental factors account for the remaining 30–70. Thus, any alterations to egg characteristics necessitates the consideration of both genetic and environmental variables [5].

Light affects the mood, physical health, and productivity of chickens. By carefully regulating the spectral properties of LED light bulbs during brooding and raising periods, caged layer chickens could experience improvement in immunological efficiency, bone growth, and production effectiveness. These benefits are observed when considering a combination of light properties, such as sources, intensity, color, and photoperiod routine [6].

Increased lighting intensity may enhance bone growth in birds by facilitating increased exercise through activities such as leaping and flying [7]. Furthermore, a study observed reduced ionized Calcium (Ca) concentrations and higher bone formation indicators in Lohmann Brown Lite pullets raised under 50 lx for 8 weeks compared to those maintained under 10 lx. This suggests that calcium homeostasis and bone development in young Lohmann Brown Lite chickens may benefit from increased light intensity [8].

Unlike traditional chickens, modern hybrid hens lay more than 320 eggs annually. Chickens require a large amount of calcium (Ca) for egg shell formation, which constitutes 10% of the egg weight, with Ca accounting for 40% of its composition [9]. The eggshell serves as a barrier to prevent damage, safeguarding the eggs’ interior from the time of oviposition until consumption. However, in the later stages of the production cycle, as laying hens age (beyond 60 weeks or more), there is a noticeable drop in both egg and shell quality. This decline may be attributed to several factors, including the increase in egg size, decline in nutritional metabolism (particularly Ca), and an elevation in reproductive hormones (particularly estrogens) that occurs at the end of the production cycle [10, 11].

In birds, vitamin D is crucial for the metabolism of Ca and phosphorus (P). The chicken industry has long relied on vitamin D3 as it is a readily available form of the vitamin. Egg production is an extremely regulated process that depends on the availability of specific nutrients at specific periods during a 24-hour cycle. Calcium, an essential nutrient, constitutes 40% of eggshells. Moreover, Ca contributes to healthy eggshells and strong bones [12].

Supplemental vitamin D3 was most often detected in patients treated with cholecalciferol. Once absorbed by the intestine, cholecalciferol travels to the liver, where it undergoes 25-hydroxylation to form 25-hydroxycholecalciferol (25OHD3), after which it undergoes carbon 1-hydroxylation in the kidney to form 1,25-dihydroxycholecalciferol (1-25OHD3) [13].

## Materials and method

### Birds and management

The experiment was conducted at the Middle East Agricultural Company Ltd., Al-Najaf Province, southern Iraq. Two rearing houses (control and treatment), each housed 75000 Lohmann Brown Classic chicks that were reared in open-sided rearing cages from one day old until they reached 18 weeks of age. Subsequently, they were transferred to laying houses, where each cage (100×40×45 cm^3^) accommodated eight hens (500 cm^2^/bird), one feeder, and two nipples. This housing arrangement persisted until the end of the experiment, when the hens reached 89 weeks of age. Throughout the laying period, the hens were subjected to a specific light regimen (light = 14 h; dark = 10 h a day). Egg counting, vaccination, daily feed consumption, drinking water, manure cleaning, temperature, and ventilation in the rearing and laying houses were controlled using the ORION computer system to to ensure compliance with the guide’s specifications [14].

After 19 weeks of being raised under identical conditions (in both treatment and control houses), the chickens in the treatment group were transferred to laying houses with a target daily temperature of 21˚C and given a diet similar to that of the control birds.

### Laying performance

Daily data were recorded, encompassing parameters such as laying rate, feed consumption, feed conversion ratio (FCR) per kilogram and by dozens of eggs, egg production rate per hen per day (HD), egg mass, egg weight, and mortality rate. Alterations in body weight were calculated by comparing the first and last measurements taken at each time point.

### Egg quality measurements

Every egg laid was weighed and evaluated, and data on mortality, cracked eggs, and shell-less eggs was documented daily per specific house (European Economic Community, 1989). In addition, the eggs were graded as extra-large (> 73 g), large (73–63 g), medium (63–53 g), or small (< 53 g).

At 75 weeks of age, 100 eggs were randomly sampled from each to determine the average egg quality at that time point. These eggs were individually weighed and examined using a multitester device (QCM-System, TSS, York, UK) to evaluate both the shell and internal material quality. Additionally, for each house, the shape index of the eggs was determined for each replicate, their length and width were measured, and the external and internal content qualities of eggs were evaluated, including shell and content weight, dimensional measures, and rupture force. Furthermore, percentages of yolk and albumin, yolk index, and Haugh units (HU), were calculated [15].

### Analysis of experimental diets

The diets used in this study were formulated according to the nutritional requirements of the “Lohmann Brown-Classic Layer,” which were either in line with or exceeded the recommendations of the NRC (1994) [16]. From 19 weeks of age onwards, the diet was supplemented in three phases: 19–50 weeks, 50–70 weeks, and > 70 weeks, with protein contents of 16.10%, 15.6%, and 14.8%, respectively. The diet contained 2700–2750 metabolized energy\kg. Each bird received a daily feed allowance of 115 g, as shown in (Table 1).

**Table 1:** Ingredient composition of the experimental diets (% per fed basis, unless declared elsewhere).

Ingredient	Proportion (%) in diet
**Soybean meal**	21.10
**Maize**	59.7
**Wheat Bran**	3.8
**Veg. oil**	1.70
**Lime Stone**	10.70
**NaCl**	0.30
**Lysin**	0.30
**Methionine**	0.40
**Premix** ^**1**^	2.00
**Calculated Analysis**	
**ME MJ/Kg**	11.20
**Crude Protein**	16.00%
**Crude Fat**	3.00%
**Fiber**	3.00%
**Linoleic acid**	1.10%
**Lysine**	0.75%
**Methionine**	0.36%
**Met±Cys**	0.60%
**Arginine**	0.70%
**Cystine**	0.25%
**Isoleucine**	0.53%
**Tryptophan**	1.40%
**Threonine**	0.40%
**Valine**	0.53%
**Calcium**	3.6%
**Phosphorus(total)**	0.55%
**P. Available**	0.5%

1 the Belgium origin (trouw nutrition) premix components were inserted in the diets (per kg of diet): crude protein 3.1%, digestible lysine 1%, digestible methionine 4.4%, digestible methionine ± cystine 5.1%, digestible threonine 0.9%, digestible tryptophane 0.14%, calcium equivalent 30.9%, digestible phosphorus 10%, sodium 5.5%, Vitamin A400000 IU/kg, Vitamin D3 100000 IU/kg, vitamin E equivalent 1800IU/kg, Vitamin K 80ppm, Vitamin B1 80ppm, Vitamin B2 160ppm, pantothenic acid 435ppm, niacin amide 1200ppm, Biotin 1600ppb, B12 1200ppb, folic acid 40ppm, B6 160ppm, Vitamin C 4000ppm, Betaine 4000ppm, Manganese 2800ppm, Iodine 40ppm, selenium 5ppm, phytase 30000 ftu/kg, Danisco xylanase 50000u/kg, iron 2800ppm, copper 600ppm, zinc 2400ppm

### Statistical analysis

Data were investigated using SPSS version 21. The results were presented as mean ± standard errors (SE), and a P value < 0.05 was considered statistically significant. The LSD was used to find differences between the groups. The data were statistically analyzed using the one-way ANOVA.

### Experimental design

The experiment was designed to determine the differences between the control and treatment groups in terms of light durations and intensities, limestone ratio, and different doses of vitamins, as shown in Table 2.

**Table 2:** The differences in feed consumption, light duration and intensity, limestone ratio, and different vitamin doses between the control and treatment groups.

**Control Group (all parameters as recommended by company guide**	**Age (Weeks)**	**Feed Consumption (g)/bird/day**	**Intensity of Light (yellow color) Exposure (lux\m** ^**2**^ **)**	**Duration of Light Exposure (hours)**	**Limestone Ratio Included in the diet**	**Different Doses of Vitamins Supplementation**
	20–29	115	10–15	13–14	3.6%	AD3E (5 days)
	30–39	115	10–15	14	3.6%	AD3E (5 days)
	40–49	115	10–15	14	3.6%	AD3E (5 days)
	50–59	115	10–15	14	3.8%	AD3E (5 days)
	60–69	115	10–15	14	3.8%	AD3E (5 days)
	70–79	115	10–15	14	3.9%	AD3E (5 days)
	80–89	115	10–15	14	3.9%	AD3E (5 days)
**Treatment group**	**Age (Weeks)**	**Feed Consumption (g)/bird/day**	**Intensity of Light (X color) Exposure (lux\m** ^**2**^ **)**	**Duration of Light Exposure (hours)**	**Limestone ratio Included in the diet**	**Different Doses of Vitamin Doses**
	20–29	112**	10–15	13–14 + 5 minutes	3.7%	AD3E 2 times/day (5 days)
	30–39	112**	17	14 + 8 minutes	3.8%	AD3E 2 times/day (5 days)
	40–49	112**	17	14 + 8 minutes	L3	AD3E 2 times/day (5 days)
	50–59	112**	Y4	Z4	L4	AD3E 2 times/day (5 days)
	60–69	112**	Y5	Z5	L5	AD3E 2 times/day (5 days)
	70–79	112**	Y6	Z6	L6	AD3E 2 times/day (5 days)
	80–89	112**	Y7	Z7	L7	AD3E 2 times/day (5 days)

### Ethical approval

Ethical approval was not required for this study. However, all birds in the current study were treated humanely according to the International and National criteria of animal care and use.

## Results

### Feed consumption and egg production rate

The daily average feed intake was unaffected by the varying limestone ratios given to the treatment group and by the different light intensity exposures, remaining consistent at 115 g for the control group and 112 g for the treatment group. However, despite this consistency, the treatment group exhibited a significantly lower feed consumption (*p*<0.01) compared to the control group.

A significant (*p*<0.05) interaction was observed between the level of limestone intake, ranging from the baseline level of 3.6% in the control and 3.7% in the treatment group (with the same light intensity and an additional duration of 5 minutes). Notably, an increase in the egg production rate of 0.9% was recorded until 29 weeks of age; however, this change was not significant (*p*<0.05). Subsequently, with regard to egg production, from to 30–39 weeks of age, the treatment group provided with a 3.8% limestone intake and higher light intensity (17 Lux/M^2^) and duration of exposure (8 minutes more), recorded a significant (*p*<0.05) 1.4% increase compared to the control group. From 40–49 weeks of age, the treatment group provided with limestone level L3 and higher light intensity (17 Lux/M^2^) and duration of exposure (8 minutes more) recorded a significant (*p*<0.01) 1.9% increase compared to the control group. From 50–59 weeks of age, the treatment group subjected to limestone level L4, and light levels Y4 and Z4 exhibited a significant (*p*<0.0001) 3% increase compared to the control group. From 60–69 weeks of age, the treatment group subjected to limestone level L5, and light levels Y5 and Z5 exhibited a significant (*p*<0.00000001) 5.8% increase in compared to the control group. In addition, from 70–79 weeks of age, the treatment group subjected to limestone level L6, and light levels Y6 and Z6 exhibited a significant (*p*<0.00000001) 8.6% increase compared to the control group. However, the highest increase was observed from 80–89 weeks of age, which were subjected to limestone level L7, and light levels Y7 and Z7, and (V6) showed a significant (*p*<0.000000003) 9.6% increase compared to the control group (Table 3).

**Table 3:** The egg production, body weight, and other egg characteristics of the control and treatment groups.

Control Group Age\ Weeks	Egg production rate(hen/day) % with age/ weeks	Body Weight/g	FCR/Body Weight	FCR/Egg	Egg Weight/g	Egg mass/g/H.D- week
**20**–**29**	10–95	1830±30.8	69.73±29.35	1.45	55.63	47
**30–39**	94.2–93	1937.5±2.40	327.94±23.57	1.22	62.51	58.68
**40**–**49**	93–90.5	1962±2.34	462.13±89.05	1.25	64.75	59.54
**50–59**	90.9–86	1962.7±2.34	356.28±66.44	1.23	66.13	58.40
**60–69**	86–81	2012.50±3.03	388.45±86.93	1.38	67.10	55.95
**70–79**	80–75	2043±2.55	372.67±82.24	1.48	67.76	52.37
**80–89**	74–68.9	2065±1.65	551.56±81.99	1.61	68.21	48.76
**Treatment Group. Age / weeks**	**Egg production rate(hen/day) %with age/ weeks**	**Body Weight/g**	**FCR/ Body Weight**	**FCR / Egg**	**Egg Weight/g**	**Egg mass/g/H.D- week**
**20**–**29**	10–96	1826 ±3.6	68.53±28.85	1.41	55.99	47.88
Significance	NS	NS	NS	NS	NS	NS
**30**–**39**	95.2–95	1933.5±2.44	316.54±21.54	1.18	62.77	59.85
Significance	S*	NS	NS	S*******	NS	S***
**40**–**49**	95–92.7	1957±2.59	336.93±61.59	1.20	65.09	61.03
Significance	S**	NS	NS	S********	NS	S******
**50**–**59**	92–91	1957.5±2.59	541.98±101.5	1.30	66.49	60.71
Significance	S****	NS	NS	S******	S*	S*******
**60**–**69**	90–87.5	2007.60±2.42	322.27±23.15	1.26	67.63	60.33
Significance	S*******	NS	NS	S********	S**	S********
**70**–**79**	87.5–84	2033±2.54	307.63±21.96	1.31	68.46	58.80
Significance	S*******	S**	NS	S********	S***	S********
**80**–**89**	83–78.5	2050±1.07	732.44±58.58	1.39	69.23	56.14
Significance	S********	S***	NS	S********	S****	S********

*: (*p*<0.05)

**: (*p*<0.01)

***: (*p*<0.001)

****: (*p*<0.0001)

*******: (*p*<0.0000001)

********: (*p*<0.00000001)

### Body weight and egg characteristics

No significant (*p*>0.05) difference in body weight was observed between the treatment and control groups from 20–69 weeks of age. However, from 70–79 to weeks of age, the treatment group exhibited a significantly (*p*<0.01) lower body weight compared to the control (2045 g vs 2055 g, respectively). Similarly, from to 80–89 weeks of age, the treatment group exhibited a significantly (*p*<0.001) lower body weight compared to the control (2057 g vs 2073 g, respectively).

### FCR/ weight or egg

The FCR/body weight change was not significant (*p*˃0.05) between the treatment and control groups across all the weeks. Additionally, the FCR/egg exhibited no significant differences (*p*˃0.05) between the treatment and control groups at 20–29 weeks of age. However, at weeks 30–39, 40–49, and 50–59, the treatment group demonstrated a significant increase (*p*<0.00000001) in FCR/egg compared to the control group (1.18 vs 1.22, 1.20 vs 1.25 and 130 vs 1.38). The most significant increase (*p*<0.00000001) was observed at weeks 80–89, with FCR/egg values of 1.39 in the treatment group vs 1.61 in the control group.

### Egg weight

There were no significant (*p*>0.05) differences in egg weight between the treatment and control groups from 20–49 weeks. However, from 50–59, 60–69, 70–79, and 80–89 weeks, the treatment group exhibited significantly higher egg weights (*p*<0.0001) compared to the control group (66.49 vs 66.13, 67.63 vs 67.10, 68.46 vs 67.76, and 69.23 vs 68.21, respectively).

### Egg mass

Egg mass did not show any not significant (*p*>0.05) alterations between the treatment and control groups at 20–29 weeks. However, at 30–39, 40–49, and 50–59 weeks, the treatment group exhibited significantly higher egg mass (*p*<0.0000001) compared to the control group (59.85, 58.68, 61.03, 59.54, and 60.71 vs 58.40), The most significant increases (*p*<0.00000001) were observed at weeks 60–69, 70–79, and 80–89 between the treatment group and the control group (60.33, 55.95, 58.80, 52.37, and 56.14 vs 48.76).

### Mean of egg weight ratios

At 60–90 weeks, the treatment group exhibited significantly less (*p*<0.05) mean egg weights in eggs grades 53 g or lesser compared to the control group. Conversely, for eggs graded 63–73 g, the treatment group exhibited a higher mean egg weight compared to the control group (55.08 vs 51.37; *p*<0.05). However, the differences in the mean egg weights among the other egg groups were not significant (*p*˃0.05), as shown in Table 4.

**Table 4:** Influence of calcium, light, and feed levels on egg weights ratios.

60–90 weeks age	Control group production %	Treatment group production %	Significance
53 g and less	7.24±0.40	4.37±0.32	S*
53–63 g	36.77±0.73	34.47±0.90	NS
63–73 g	51.37±0.73	55.08±1.1	S*
73 g and more	4.61±0.77	6.07±0.36	NS

*: (*p*<0.05)

### Egg characteristics

At 75 weeks, the albumin weight, yolk weight, yolk diameter, shape index, and HU were not significantly different (*p*˃0.05) between the treatment and control groups. However, significant differences were observed in the treatment group compared to the control group for shell thickness (0.352 vs. 0.344; *p*<0.01) and shell weight (9.03 vs 8.79; *p*<0.0001), as shown in Table 5.

**Table 5:** Influence of feed consumption, calcium, and light on egg characteristics.

75 weeks age	Control group	Treatment group	Significance
**Albumin weight (g)**	41.95±0.037	42.016±0.036	NS
**Yolk Weight (g)**	15.90±0.014	15.93±0.016	NS
**Yolk Diameter**	3.02±0.02	3.10±0.04	NS
**Shell thickness (mm)**	0. 344±0. 042	0. 352±0. 041	S**
**Shape Index**	1.33±0.05	1.34±0.06	NS
**Shell weight (g)**	8.79±0.007	9.03±0.013	S****
**HU**	79.55±0.05	79.71±0.08	NS

**:(*p*<0.01)

****:(*p*<0.0001)

### Broken and shell-less egg percentage

The percentages of broken and shell-less eggs were not significantly (*p*˃0.05) impacted at phase 1 between the treatment and control groups. However, the treatment group showed a significant decrease in phases 2 (*p*<0.00001) and 3 (*p*<0.000001) compared to the control group (0.715 vs. 0.946 and 0.86 vs 1.55, respectively). Additionally, shell strength was significantly (*p*<0.00001) increased across all the phases of treatment group compared to the control group (41.80 vs 38.40), as shown in Table 6.

**Table 6:** Influence of feed consumption, calcium, and light on the percentages of broken and shell-less eggs.

Weeks of age	Control group	Treatment group	Significance
**Broken and shell-less percentage phase 1**	0.555±0.009	0.529± 0.017	NS
**Broken and shell-less percentage phase 2**	0.946±0.010	0.715± 0.011	S*****
**Broken and shell-less percentage phase 3**	1.55±0.050	0.86±0.014	S******
**Shell strength /Newton**	38.40±0.49	41.80± 0.35	S*****

*****: (*p*<0.00001)

******: (*p*<0.000001)

## Discussion

The improvements in chicken performance is considered a major achievement in the poultry industry because as the hen reaches the end of its peak production ends, it lays fewer eggs and of poorer quality. This decline can be attributed to various factors, including a decrease in breeder hen vitellogenesis, lipogenesis, antioxidant, and immunological status, alongside potential issues such as poor management, improper feeding, genetic predisposition, or exposure to a toxic environment [17].

In the present study, feed restriction (112 g vs. 115 g) potentially affected feed consumption and egg production in the treatment group. These observations align with those of [18], which demonstrated that a feed limitation of 10% improved the laying performance, possibly due to enhanced maintenance processes regulated by body weight to prevent overeating [19, 20]. Notably, in this study, the feed reduction was less than 3% resulted in savings of over 55 tons of food at a price of 33,000 USD.

Supplementation of 3.8% limestone intake with a light intensity of 10–15 Lux/M^2^ for a duration of 14 h + 5 min in the treatment group did not significantly (*p*<0.05) increase the egg production rate by 0.9% until 29 weeks of age. However, from week 30–39, the treatment group with limestone intake of 3.9% and light intensity 17 Lux/M^2^ and duration of 14 h + 8 min, combined with two doses of vitamin AD3E, exhibited a significant (*p*<0.05) 1.4% increase compared to the control group. By the end of the experiment, the treatment groups with the limestone intake parameter L7, and light conditions Y7 and Z7 recorded a significance (*p*<0.000000003) 9.6% increase in egg production compared to the control group that was reared according to the guide’s specifications.

The results obtained in this study represent a pioneering achievement in the field, as they demonstrate the combined effects of limestone, light, and vitamin supplementation on egg production. Interestingly, previous research has indicated varying outcomes regarding the influence of calcium intake on egg production in chickens. For instance, some studies have shown that increasing Ca intake from 3.68 to 4.26 g/d (3.22 to 3.83% of the diet) did not affect egg production in chickens aged 55–70 weeks [21]. Similarly, another study reported that in chickens aged between 24–27 weeks, neither egg production nor egg weight was influenced by the increase in Ca intake from 3.58 to 4.35 g/d (3.3 to 4.1% of the diet [22]. Moreover, a study that examined chickens aged 38–62 weeks reported that increasing the Ca component of the feed from 3.85 to 4.40 g/d (3.5 to 4.0% of the diet) had no effect on the egg production, egg weight, or FCR [23].

Conversely, in 21-week-old W36-Hyline hens, an elevation in Ca intake (from 2.1 to 4.2 g of Ca/b/d) at a fixed dietary dose of available phosphorous (P) (0.40%) was observed to boost egg production characteristics, as reported by [24]. Similarly, increases in both egg production and egg quality characteristics were observed when the Ca:P ratio was maintained at 12:1 (with a Ca consumption of 4.1 to 4.6 g of Ca/b/d). Additionally, both [25, 26] reported that giving both young and grown chickens a Ca supply in the form of large pieces (0.8–2 mm) of limestone boosted both egg weight and production. Consequently, the external and internal quality characteristics of eggs produced before and after the age of 39 weeks exhibit significant changes [27].

In the present study, the observed increase in egg production may be attributed, in part, to the varying light intensities applied to hens from ranging from 10 Lux/M^2^ to Y7. These results align with those of [28], which suggest that light properties play a role in managing egg quality. Moreover, light sources [29], light strength [30], and the homogeneity of light distribution [31] have been reported to affect the laying productivity of chickens.

Consistently, the inclusion of vitamin supplementation in the drinking water of the control group, in addition to rations, was sound to be crucial in maintaining with the productivity of hens. These findings align with those of [32], which observed significant increases in egg production, egg mass, fertility, and hatchability of eggs with the inclusion of vitamin A supplementation in the diet of layer. Similarly [33], the reported that the addition of vitamin E to the diet improved both egg production and quality. Additionally [34], attributed recorded increases in both egg production and quality to the early and long-term utilization of vitamin D.

Notably, in this study, the addition of different doses of vitamins across all experimental periods resulted in increased egg production.

There was no significant (*p*>0.05) difference in body weight between the groups from 20–69 weeks, likely due to the feed restriction of less than 3% in the treatment group. However, the differences in weight were obvious from 70–79 and 80–89 weeks, with the treatment group recording significantly lower weights (*p*<0.01 and *p*<0.001, respectively). This could be attributed to the cumulative effect of reduced feeding over long periods, preventing overweight conditions and maintaining correct body weight [19]. Moreover, the FCR/egg ratio remained relatively unchanged from to 20–29 weeks, but a significant difference (*p*<0.0000001) emerged from 30–90 to weeks in the treatment group, indicating conserved high egg production despite lower feed consumption. These results align with those of [18, 35, 36], which demonstrated that a feed limitation of 10% improved laying performance, by aiding in the preservation of proper body weight and preventing overeating.

These results were in partial disagreement with [18, 36], as they advocated for minimizing feed consumption by more than 20 or 30 percent of *ad libitum*, whereas this study minimized feed consumption by less than 3%.

Despite the increases in egg weight and egg mass, no significant (*p*>0.05) differences were found between the two groups from 20–29 weeks. However, from 30–89 weeks, the treatment group showed gradual and significant increases, attributed to the combination of all the treatment parameters (many doses of limestone, many types of light intensity, and many doses of vitamins), consistent with those reported by [25, 26].

The treatment group recorded a significantly (*p*<0.05) lower mean egg weight than the control group from weeks 60–90 in the category of eggs weighing 53 g and lesser, while it was higher in the category of eggs weighing 63–73 g. However, the differences among the other categories were not significant. These findings indicate a favourable profit margin and the most desirable egg size (63–73 g) for human consumption. This aligns with the results reported by [37–39], which demonstrated that hens reared in furnished cages with different grades of limestone and Vitamin D under an intermittent light regime had better eggshell quality and egg weight.

Although the albumin weight, yolk weight, yolk diameter, and HU were higher in the treatment group than those in the control group, the increases were not significant at 75 weeks. However, the shell weight and shell thickness of the treatment group showed significantly higher (*p*<0.0001 and *p*<0.01) differences. The findings align with those of [40], which demonstrated that enhanced scotophase increased the Ca and P content in the eggshell and improved eggshell hardness. Similarly [25, 41], indicated that dietary Ca and P and levels influenced eggshell quality throughout the laying phase to guarantee eggshell quality in the final third of the production period. However, when brown-egg-laying chickens were fed [42] a Ca intake of more than 3.4 g/d, eggshell deformation was not affected. The Ca was delivered in the form of limestone. Therefore, variations in strain, age, egg output, and nutritional configurations of the diets may account for the inconsistency among studies regarding Ca requirements and eggshell quality.

The increase in the proportion of broken and shell-less eggs between the two groups was not statistically significant throughout the first phase of the study. However, significant improvement was observed in the treatment group over the course of phases 2 and 3, with the latter showing an increase in outcomes with a P-value less than 0.000001. These results seem to align with [12, 25], which demonstrated that an increase in Ca consumption enhanced shell weight, thickness, and density, and decreased the proportion of broken and shell-less eggs. Notably, the last stage of the egg production exhibited the greatest increase in shell weight and density.

Since egg production in laying hens is directly related to the nutrients they receive in their feed, changes in diet formulation may help the poultry industry achieve its ultimate aims. Producers prioritize diet quality and composition because feed accounts for 70–80% of the total cost of egg production [43]. Least-cost linear programming can be utilized to create a diet that is inexpensive while satisfying the birds’ nutritional requirement. While individual businesses and companies may have differing priorities, most commercial enterprises strive to maximize revenue by optimizing their inputs and outputs. The formulation of poultry diets and decision-making processes can benefit from the rapid development of big data and communication technologies [44]. Weekly live performance evaluations, the total egg yield per house, and the treatment expenses for each house were used for financial evaluation. The expected profits from selling eggs served as the basis for calculating the gross margin (30-dozen-egg package). As all treatments followed the same management and feeding protocols and had access to the same housing and infrastructure, there was no difference in labor allocation. Consequently, the economic analysis excluded such expenses.

## Conclusion

The innovative nutritional protocol outlined in this study decreased the final feed consumption of the treatment group by more than 55 tons, resulting in saving equivalent to 33,000 USD. Moreover, the increase in egg production within the treatment group amounted to more than 1,425,408 eggs compared to the control group and the strain’s standard guidelines over 490 days of production, equivalent to 142,541 USD. At the same time, there was a significant decrease in light color and shell-less eggs in the treatment group, contributing as addition 5000 USD in profit.

Finally, the treatment group yielded a total revenue of 180,541 USD, surpassing that of the control group (with profit exceeding the control group’s baseline profit).

This study represents a pioneering effort worldwide, conducted in two houses under field circumstances, each housing 75,000 layers for 89 weeks. These results signify significant achievements in the poultry industry, demonstrating excellent revenue and advancements.
